# Therapeutic Advances in Advanced Basal Cell Carcinoma

**DOI:** 10.3390/cancers16173075

**Published:** 2024-09-04

**Authors:** Samer Alkassis, Maya Shatta, Deborah J. Wong

**Affiliations:** 1 Division of Hematology/Oncology, Department of Medicine, University of California, Los Angeles, CA 90095, USA; 2 Covenant Health-Statcare Hospitalist Group, Knoxville, TN 37919, USA

**Keywords:** hedgehog inhibitor, anti-PD1, checkpoint inhibitor, basal cell carcinoma

## Abstract

**Simple Summary:**

Basal cell carcinoma (BCC) is the most common form of skin cancer. In advanced stage, BCC exhibits aggressive behavior, leading to local tissue destruction. Thus, a multidisciplinary approach and specialized treatment strategies are required for effective disease management and improvement of the patient quality of life. For low-risk disease, surgical intervention is the modality of choice while radiotherapy is indicated in unresectable tumor, positive margins post-surgery when re-excision is not feasible, and in the presence of high-risk features for recurrence. For locally advanced and metastatic disease, the emergence of systemic treatments including hedgehog inhibitors and immune checkpoint inhibitors has significantly improved the outcomes. Nonetheless, developing resistance to therapy presents a significant challenge in management. Consequently, development of novel therapeutic strategies is ongoing to address improving treatment efficacy.

**Abstract:**

Basal cell carcinoma (BCC) is the most common type of cancer with an estimated 3.6 million cases diagnosed annually in the US alone. While most cases are treatable with low recurrence rates, 1–10% progress to an advanced stage which can behave aggressively, leading to local destruction and posing substantial challenges in management. The pathogenesis often involves dysregulation of the patched/hedgehog protein family, a pivotal pathway targeted by recently approved therapies. Furthermore, the role of immunotherapy is evolving in this type of tumor as we learn more about tumor microenvironment dynamics. In recent years, there have been advancements in the therapeutic landscape of advanced BCC, offering patients new hope and options for managing this complex and potentially life-threatening condition. In this review, we aim to provide a comprehensive overview of this disease, including the risk factors, underlying pathogenesis, current treatment options of advanced disease, and the ongoing exploration and development of novel therapies.

## 1. Introduction

Basal cell carcinoma (BCC), also known as keratinocyte tumor, is a non-melanomatous skin cancer originating from the totipotent cells of the hair follicle and is the most common type of cancer [1]. Due to low mortality rate, there is no cancer registry that collects data on BCC [2]. According to the skin cancer foundation, 3.6 million cases are diagnosed each year in the US alone [3]. The incidence of BCC increases after 40 years of age and occurs more commonly among men [4]. However, given the increased UV exposure naturally or artificially lately, there has been an increase in its incidence among the younger population, particularly in women [5,6]. 

BCC can progress to advanced cases in 1–10% [7]. Further classification of advanced disease includes locally advanced (la-BCC) or metastatic (mBCC) [8]. Although there is no consensus about la-BCC definition, these tumors are generally large and aggressive for which surgery would be disfiguring or associated with loss of function [9,10]. Radiation is often offered in these cases if the radiation field can encompass the locoregional area of BCC involvement but does not address distant metastatic disease. Metastatic BCC has an incidence rate of 0.0028% to 0.55% [11], with the most common sites including regional lymph nodes followed by hematogenous spread to lungs and bones [12]. Even in the absence of distant metastases, however, BCC can behave aggressively and be fatal due to local destruction [13,14]. Therefore, advanced BCC often requires a multidisciplinary approach and specialized treatment strategies to effectively control and improve the patient’s quality of life [14]. In recent years, there have been advancements in the treatment of advanced BCC, offering patients new hope and options for managing this complex and potentially life-threatening condition. This article will provide an overview of advanced BCC including the risk factors, pathogenesis, and the latest developments in treatment of locally advanced, unresectable, and metastatic BCC.

## 2. Risk Factors and Subtypes

DNA mutation caused by ultraviolet radiation B (UVB), in particular, is the most common risk factor for BCC [1]. Unrepaired DNA damage including DNA strand breaks and crosslinks can lead to cutaneous carcinogenesis by transforming into genetic mutations [5,15]. In addition, oxidative stress as well as suppression of antitumor immunity from UVB exposure play a significant role in BCC development [16,17]. Age-related factors including genomic instability, impaired DNA repair, and chronic inflammation provide a suitable microenvironment for tumor growth [16,18,19]. Other risk factors include skin phototype (Fitzpatrick I and II), genetic syndromes, immunosuppression, carcinogenic substances (e.g., arsenic), and radiation [20,21]. Immunosuppression in patients with organ transplant and HIV seropositivity increased the incidence of BCC by 10- and 2-fold, respectively [22,23]. Exposure to ionizing radiation in a different setting (i.e., therapeutic, environmental) is associated with a higher risk for BCC, more commonly with the infiltrative subtype [16,24].

The distinct clinical types of BCC are classified according to the histopathological diagnosis in addition to their invasive behavior and recurrence risk [20]. Low-risk subtypes include nodular (60–80%), superficial multicentric (20%), pigmented, cystic, and fibroepithelial (Pinkus). Superficial and nodular subtypes are the most commonly encountered in clinical practice [25]. High-risk subtypes include metatypical, morphoeiform (sclerosing, desmoplastic), infiltrative, BCC with sarcomatoid differentiation, and micronodular [26]. Metatypical BCC, also known as basosquamous carcinoma, is characterized by the presence of overlapping histologic between BCC and squamous cell carcinoma [27].

## 3. Pathogenesis and Genetics

**Figure 1:**
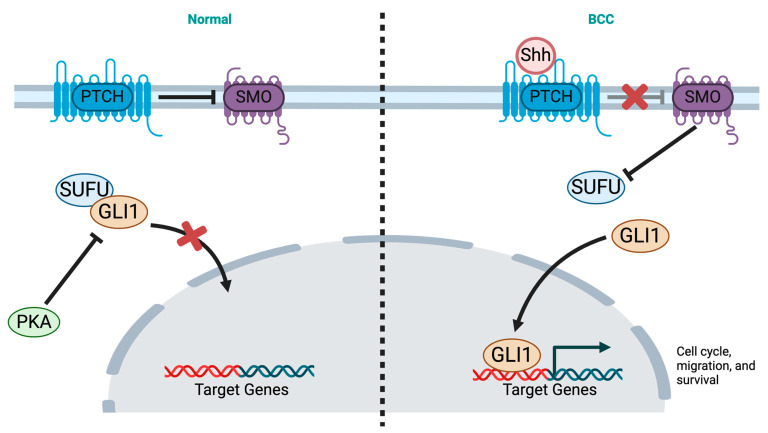
Genetics of BCC. Left (normal): PTCH1 suppresses SMO in the absence of HH ligands. SUFU and PKA are negative regulators of GLI. Right (BCC): Shh ligands bind to PTCH1 → SMO is de-repressed and activates the GLI family of transcription factors which are translocated to the nucleus after dissociating from their inhibitor SUFU. GLI targets genes involved in cell cycle and survival. GLI: Glioma-associated oncogene, Shh: Sonic Hedgehog, PKA: Protein Kinase A, PTCH1: PATCHED1, SMO: Smoothened protein, SUFU: Suppressor of Fused. Adapted from (Hedgehog Signaling Pathway), by BioRender (2024).

The pathophysiology of BCC involves an interaction of genetic factors, sporadic somatic mutations, and environmental factors [1,28]. One of the most commonly implicated genes in the pathogenesis of BCC is the patched/hedgehog protein family (HH) [29]. Patched receptor (PTCH) is a transmembrane protein that represses HH signaling cascade by inhibiting smoothened (SMO) protein [29]. Upon binding to HH ligand, PTCH is inactivated, leading to cell cycle dysregulation. PTCH suppression causes the release of SMO and activation of glioma-associated oncogene (GLI) proteins which are translocated into the nucleus after dissociating from their inhibitor suppressor of fused (SUFU). GLI proteins serve as transcription factors in the nucleus for cell proliferation and thus an active pathway [29,30] (Figure 1). Mutations in the HH pathway have been identified; somatic PTCH1 mutations causing loss of function were found in up to 75% of cases, whereas activating mutations of SMO were identified in 10–20% [31]. Less common mutations of this pathway included loss of function of the SUFU, which is a negative regulator of the hedgehog (HH) pathway, and mutations of the PTCH1-homologue PTCH2 [31,32].

Gene polymorphisms and some genetic disorders are associated with high risk for developing BCC [28]. Such syndromes include but are not limited to Nevoid Basal Cell Carcinoma Syndrome (NBCCS), also known as Gorlin syndrome, Cowden syndrome, Bazex–Dupre–Christol syndrome (BDCS), and Xeroderma pigmentosum [1,28,30]. Genetic alterations of TP53 have also been implicated [31]. These mutations are most commonly caused by UV radiation, causing what is known as UV-induced mutagenesis, and can occur sporadically in 61% of cases. Another identified pathway includes the Hippo–Yap, which is essential for restricting tissue growth; nuclear YAP expression has been identified in BCC [1,31]. In human cancers, increased telomerase activity is one of the primary characteristics identified. UV-induced mutagenesis of the TERT gene which maintains the length of telomere is commonly reported in BCC with a reported frequency of 39–74% [33]. UV-induced mutagenesis of DPH3-OXNAD1 which is a bidirectional promoter presents in 42% of tumors [34]. UV radiation can also induce inflammatory responses and contribute to the pathogenesis of BCC by affecting toll-like receptors, ERBB2, NLRP3, cyclooxygenases, and many more [5,35]. A study showed that an association between arsenic exposure and risk of BCC can be influenced by genetic factors, including variants of the AS3MT gene [21].

## 4. Tumor Microenvironment in Advanced BCC

The tumor microenvironment (TME) is a network of cells and molecules that interact with cancer cells within a tumor. It plays a very important role in tumor growth, progression, and response to treatment [36,37]. Understanding the TME is an important area of research in cancer management. In BCC, the microenvironment consists mainly of T-helper 2 cytokines [37], and T cells that are antitumorigenic are downregulated by signaling from cancer-associated fibroblasts (CAFs) and regulatory T cells (Tregs) [38]. An immunosuppressive TME is usually the result of an interplay between TGF-beta inducing Tregs into the TME and the myofibroblastic differentiation of fibroblasts [36,39]. Other chemokines including IL-6, IL-10, and CCL22 also play a role in BCC TME. IL-6, a pro-oncogenic cytokine, promotes angiogenesis and enhances antiapoptotic activity [38]. IL-10 and CCL22 are responsible for Treg development and chemotaxis in addition to CAFs immobilizing T cells (CD4 and CD8) which have anti-oncogenic effects [40]. BCC cells can also recruit Tregs and produce IL-10, which is another mechanism in which the cancer cells contribute to an immunosuppressive TME, thus providing the ideal environment for tumor growth. Lefrançois et al. [41] characterized BCC TME by RNA sequencing. Compared to normal skin tissue, significant lymphocytic infiltration was identified. Gamma–delta T lymphocytes were higher in low-risk than in high-risk BCC, suggesting a protective effect by suppressing BCC growth. Infiltrative lymphocytes were highest in non-advanced BCC, whereas macrophage-induced inflammation was detected in advanced disease [41]. Immune checkpoint expression within BCC TME was assessed in 34 archival tissues of aggressive BCC by Deutsch et al. [42]. All the samples revealed LAG-3 and PD-1 expression in > 1% of tumor-infiltrating lymphocyte (TIL). A significant correlation between PD-1 and LAG-3 densities was also demonstrated, suggesting coordinating immunosuppression and the potential to introduced combination immunotherapy to improve clinical outcome [42].

## 5. Approved Therapies in Advanced BCC

As previously discussed, pathologic diagnosis is required to classify BCC into low- or high-risk categories. Surgical management is usually the modality of choice in patients with low-risk BCC [14]. The most common surgical procedures used include surgical excision, Moh’s micrographic surgery, electrodesiccation, and curettage, in addition to cryosurgery. Other modalities of treatment include photodynamic therapy, which is used for superficial BCC on the face and scalp, radiotherapy, and topical medications such as 5-FU and imiquimod. Indications for radiotherapy according to the National Comprehensive Cancer Network include unresectable disease, positive tumor markers after surgical resection when re-excision is not an option, and presence of high-risk features for recurrence including nodal involvement, presence of lymphovascular invasion, size > 2 cm, and aggressive subtypes (infiltrative or micronodular) or when surgical excision is associated with unacceptable morbidity or deformity [14]. For locally advanced and metastatic BCC, the emergence of systemic treatment including HH inhibitors and immune checkpoint inhibitors has significantly improved the outcomes (Table 1).

### 5.1. Hedgehog Pathway Inhibitors

#### 5.1.1. Vismodegib

**Figure 2:**
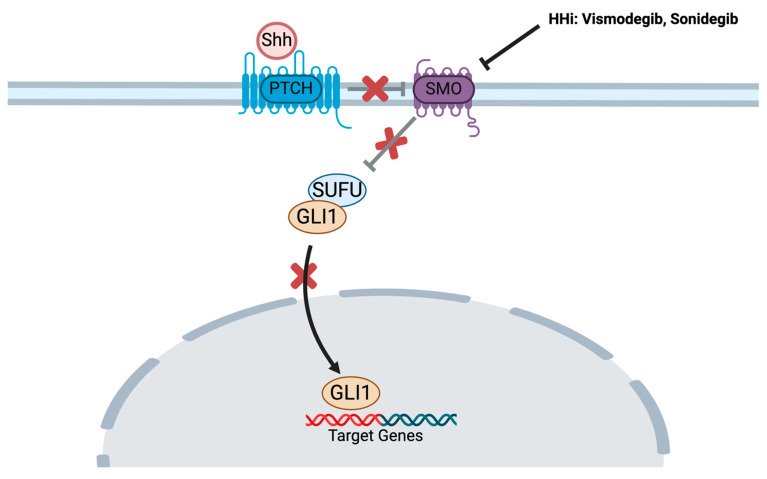
Mechanism of Action of Hedgehog Pathway Inhibitors. Hedgehog inhibitors bind to and inhibit SMO, allowing SUFU to bind to GLI1 which blocks its translocation to the nucleus. GLI: Glioma-associated oncogene, Shh: Hedgehog, HHI: Hedgehog Pathway Inhibitor, PTCH1: PATCHED1, SMO: Smoothened protein, SUFU: Suppressor of Fused. Adapted from (Hedgehog Signaling Pathway), by BioRender (2024).

BCC does not typically respond to chemotherapy [43]. Vismodegib was the first hedgehog (HH) pathway inhibitor to be approved by the United States Food and Drug Administration (FDA) in 2012 for advanced BCC [44], representing a major advance in the field. By binding to SMO, vismodegib blocks the activation of the downstream HH pathway [45] (Figure 2). The approval was based on the ERIVANCE trial [46], a phase II, multicenter, single-arm study in patients with advanced BCC who were not eligible for surgery or radiation therapy. The objective response rate (ORR) was 43% among 63 patients with locally advanced BCC (la-BCC) and 30% in 33 participants with metastatic BCC (mBCC). The most common adverse events included muscle spasms, alopecia, dysgeusia, weight loss, and fatigue [46]. Median progression-free survival (PFS) in the final analysis was 12.9 months and 9.3 months in la-BCC and mBCC, respectively. Median overall survival (OS) was 33.4 months in mBCC and had not been reached in the la-BCC population [47]. Following approval, the safety and efficacy of vismodegib were assessed in a setting representative of real-world clinical practice [48]. Included in the assessment were 1215 participants with advanced BCC (1119 la-BCC, 96 mBCC), with recurrent BCC after surgery and/radiation, or who were not candidates for surgery or radiation. The trial demonstrated an ORR of 68.5% in la-BCC and 36.9% in mBCC. Median PFS was 23.2 months and 13.1 months for la-BCC and mBCC, respectively [48]. Most patients (98%) experienced treatment-emergent adverse events (TEAE) with serious TEAE of 23.8%. The most common TEAEs were muscle spasm, alopecia, dysgeusia, asthenia, and decreased weight, and 31% of patients permanently discontinued therapy due to TEAEs. Treatment interruptions were not infrequent, occurring at a rate of 80%, and were primarily due to dysgeusia, decreased weight, and muscle spasms. Nevertheless, despite the side effects of vismodegib, in a separate study in 169 patients with locally advanced periocular BCC, vismodegib significantly improved quality of life compared to baseline [49]. In an observational study, 66 participants who received at least one dose of vismodegib were followed for duration of response as primary endpoint. ORR and disease control rate were 74.2% and 90.9%, respectively, with a median DOR of 15.9 months [50]. Another study conducted by Słowińska et al. retrospectively evaluated the data of 108 patients with advanced BCC who had received vismodegib. The median PFS (mPFS) was 30.5 months, and median OS (mOS) was 30.5 months and 41.5 months. The OS rate after 1, 2, and 3 years was 86%, 73%, and 60%, respectively [51]. Vismodegib was also studied in the neoadjuvant setting in a VISMONEO study [52]; of 55 patients with la-BCC, 44 (80%) had downstaging of the tumor after a mean of 6-month treatment of neoadjuvant vismodegib.

Despite the exciting response rates and durability of response with vismodegib, resistance to treatment has been reported [53]. One potential mechanism reported by Lefrançois et al. is decreased drug delivery to tumor, as evidenced by a high percentage of fibroblasts and adipocytes [41]. Given this fact, patients treated with vismodegib are advised to continue skin surveillance after initiation of therapy as both in-field local recurrence and development of new primary lesions can occur.

#### 5.1.2. Sonidegib

Three years after vismodegib’s approval, sonidegib was granted FDA approval in 2015 for la-BCC [54] based on the Phase II multicenter BOLT trial [55]. In this study, 230 patients with locally advanced BCC who were ineligible for curative surgery or radiation were enrolled. In a 1:2 ratio, 79 participants were randomized to sonidegib 200 mg and 151 patients to 800 mg. The ORR was 36% and 34% in the 200 mg and 800 mg groups, respectively. Dose interruption or reduction due to toxicity occurred in 32% in the 200 mg group and 60% in the 800 mg group. Side effects occurring at an incidence of 10% or higher included muscle spasms, dysgeusia, alopecia, nausea, increased creatine kinase concentration, weight decrease, and fatigue. Serious adverse events were reported in 14% and 30% in both groups, respectively. The most common reason for treatment interruption was muscle spasm [55]. At the 30-month analysis, patients with la-BCC and mBCC treated with the dosage of 200 mg daily had an ORR of 71.2% and 33%, respectively, with a median DOR of 15.7 months in la-BCC and 18.1 months in mBCC. These data supported the use of 200 mg in this setting [56]. At the final analysis (42 months), the ORR was 56% and 8% for patients with la-BCC and mBCC, respectively, treated with the 200 mg dose [57]. Similar to vismodegib, sonidegib binds to SMO, inhibiting downstream activation of the HH pathway (Figure 2).

While both vismodegib and sonidegib share many common side effects, individual patient responses can vary. Hair loss is the most common cutaneous adverse event, occurring in up to 63% and 43% of patients treated with vismodegib and sonidegib, respectively. Extracutaneous toxicity includes gastrointestinal events, with nausea reported in up to 29% with vismodegib compared to 33% with sonidegib. Dysgeusia (70.6% vs. 38% with sonidegib), muscle spasms (70.6% with vismodegib vs. 50%), and weight loss are also commonly reported [58]. Although there is limited strong evidence on how to address most of these adverse events, certain measurements can be implemented to mitigate toxicity. For instance, alopecia may be mitigated with the use of minoxidil 5% twice daily, the use of antiemetics can be effective in managing nausea, nutrition consultation can be incorporated to address dysgeusia and weight loss, while muscle relaxants, amlodipine, supplements such as L-carnitine, or physical activity may help alleviate muscle spasms.

### 5.2. Immune Checkpoint Inhibition

Similar to other solid tumors, the role of immunotherapy has emerged in advanced BCC. As of today, there are no approved immunotherapy in the frontline setting for advanced BCC [59]. Cemiplimab is the only immune checkpoint inhibitor which received FDA and EMA approval in 2021 [60] for patients with advanced BCC who were previously treated with an HH inhibitor or who are not eligible for HH inhibitor. The approval was based on a phase II, multicenter, single-arm trial in which 84 patients with la-BCC who progressed or did not tolerate HH inhibitors received cemiplimab 350 mg every 3 weeks until disease progression or unacceptable toxicity. An ORR was seen in 31% (6% complete response and 25% partial response) [61]. The final analysis of 54 patients with mBCC demonstrated ORR of 22% with 2 complete and 10 partial responses. Median PFS and OS were 10 months and 50 months, respectively [62].

Other PD1 checkpoint inhibitors have been evaluated in la- and mBCC. Pembrolizumab was investigated in a small proof-of-concept study [63]. Sixteen participants received pembrolizumab alone (9 patients) or with vismodegib (7 patients) with ORR demonstrated in 4 patients (44%) in a monotherapy group and 2 patients (29%) in a combination group. Nivolumab was also studied in 33 patients with advanced BCC after multiple lines of treatment in a phase II basket trial [64]. All patients had received HH inhibitor. Complete response, partial response, and stable disease rates were 12.5%, 18.8%, and 43.8%, respectively. A higher rate of diabetes but no thyroid dysfunction was observed, which were different from the adverse events demonstrated in the treatment of metastatic melanoma [64]. Nivolumab was also investigated as monotherapy or in combination with ipilimumab or relatlimab in 24 patients with la-BCC or mBCC [65]. Fifteen participants received nivolumab alone, eight patients received nivolumab + relatlimab, and one patient had nivolumab + ipilimumab. The ORR with nivolumab alone was 50% and 20% in the first- and second-line settings, respectively. Among 6 evaluable patients in the nivolumab + relatlimab group, 1 had progressive disease, 4 had stable disease, and 1 with partial response. No responses were seen in 5 patients with mBCC [65].

**Table 1:** Trials of Approved Therapies in Advanced BCC.

	Trial	NCT	Results
**Vismodegib**	ERIVANCE [46,47]	00833417	- ORR: 43% (la-BCC), 30% (mBCC) - mPFS: 12.9 months (la-BCC), 9.3 months (mBCC) - mOS: not reached (la-BCC), 33.4 months (mBCC)
	STEVIE [48]	01367665	- ORR: 68.5% (la-BCC), 36.9% (mBCC) - mPFS: 23.2 months (la-BCC), 13.1 months (mBCC)
**Sonidegib**	BOLT [56,57]	01327053	***30-month analysis (200 mg)***
			- ORR: 71.2% (la-BCC), 33% (mBCC)
			- Median DOR: 15.7 months (la-BCC), 18.1 months (mBCC)
			***42-month analysis (200 mg)***
			- ORR: 56% (la-BCC), 8% in mBCC
**Cemiplimab**	[61,62]	03132636	- ORR: 31% - mBCC: ORR 22%, mPFS 10 months, mOS 50 months

#### 5.2.1. Resistance to Immunotherapy

Primary and acquired resistance mechanisms to immunotherapy in BCC have been proposed in the literature. A cold tumor microenvironment, defined by the downregulation of MHC-1 molecule expression, leading to absence of infiltrating cytotoxic T-cells has been identified by Walter et al. [66]. The paucity of tumor-infiltrating CD8+ cells in addition to decreased expression of MHC-I in BCC were suggested mechanisms of resistance to adaptive immunity. Sabbatino et al. characterized similar mechanism in a patient with BCC who had resistance to frontline nivolumab [67]. Lack of β2 microglobulin, which has a significant role in MHC class I expression, was identified in addition to the absence of HLA class I antigen. Further analysis revealed PD-L1 expression < 1% [67]. Upregulation of HLA class I expression by Interferon gamma (IFNγ) released by tumor-infiltrating T cell [68] and alterations in IFNγ signaling pathways have been identified as mechanisms of resistance in other histologies [69,70]. These data in other histologies underscore the importance of MHC class I expression in response to immune checkpoint inhibition for BCC. Increased expression of immune checkpoint proteins has been identified as one mechanism of resistance in BCC. In one study published by Deutsch et al. [42], one patient who had stable disease with nivolumab (anti-PD-1) followed by partial response after combining with relatlimab (anti-LAG-3). Tissue analysis from multiple biopsies through the course of treatment showed augmented expression of LAG-3 following nivolumab therapy, suggesting coordinated immunosuppression [42]. Furthermore, the role of macrophages in ICI resistance has also been explored. Dollinger et al. compared the immune transcriptome in the tumor microenvironment in melanoma and BCC. Macrophages were over-represented in patients who did not respond to immunotherapy. However, these cells had pro-inflammatory signals, suggesting irrelevant role in resistance [71]. The immunosuppressive microenvironment, as evident by the presence of immature dendritic cells in the stroma of BCC, could explain the lack of response to immunotherapy [72]. In addition, the expression of immunosuppressive cytokines, including TGF and IL-10, has also been reported [73].

#### 5.2.2. Predictive Markers for Response to Immunotherapy

The data on biomarkers predictive of response to immunotherapy in BCC are scarce, with more investigation obtained in melanoma and other solid tumors for which immunotherapy is approved. Potential biomarkers including MHC-I expression, PD-L1 status, and tumor mutational burden (TMB) have been evaluated to predict response to ICI. Exposure to UV radiation, a known mutation, is a common risk factor for BCC. Therefore, high TMB is expected to be found in this type of tumor, leading to higher expression of neoantigen recognized by the immune system. However, despite this fact, reduced antigen presentation and lower immunogenicity have been observed [74]. This could be explained by downregulating MHC-1 and transporters associated with antigen processing-1 (TAP), which play a significant role in antigen presentation [72,74]. There has been no correlation between PD-L1 expression and the response to immunotherapy in BCC. Prior proof-of-concept study of pembrolizumab in a small sample did not find a correlation between PD-L1 expression and decrease in tumor size [63]. Stratigos et al. conducted a phase II trial to evaluate cemiplimab in 50 patients with locally advanced BCC. There was no difference objective response rate between patients who had PD-L1 expression <1% vs. ≥1% (26% vs. 27%, respectively) [75]. This was confirmed on further exploratory analysis which did not reveal significance of PD-L1 expression as a predictive response to cemiplimab [61]. The exploratory analysis by Stratigos et al. was not supportive for TMB expression in predicting response to cemiplimab. Not all patients with high TMB responded to PD-L1 blockade, whereas some with low TMB had durable responses [61]. The median TMB was 58 mutations/Mb in responders and 23 mutations/Mb in non-responders, which is significantly higher than the high TMB threshold (>10 mutations/Mb). Interestingly, some patients with high TMB and low/absent MHC-I expression did not respond to cemiplimab [61].

## 6. Ongoing Clinical Trials in Advanced BCC

Multiple trials are ongoing for advanced and metastatic BCC (Table 2). Immune checkpoint inhibitors are currently being investigated in the neoadjuvant, adjuvant, and locally advanced and metastatic settings. Combination treatment with immunotherapy and HH inhibitor is currently being explored, and other trials of novel targeted and intra-tumoral therapies are evolving.

- Cemiplimab

The role of cemiplimab in the neoadjuvant setting is under investigation for locally advanced BCC of head and neck (NCT05929664) in a Phase II trial; the primary outcomes of the trial are objective response and disease control rates. In the la- and mBCC settings, the combination of cemiplimab and sonidegib is also being explored in a single-arm, phase II trial in advanced BCC (NCT04679480). In this study, cemiplimab is administered every 3 weeks, and sonidegib is given once daily as a pulsed therapy every 4 weeks (2 weeks on, 2 weeks off). The duration of the study from screening is 32 weeks. If partial response is the best response at week 26, participants will be given the option to continue treatment with at least one of the medications until either complete response or for up to one year. The combination of cemiplimab with intra-tumoral therapy is also under evaluation. A multicenter phase 2 study will provide information whether cemiplimab, combined with intra-tumoral Vidutolimod, a TLR9 agonist, is effective in advanced, unresectable solid tumors (NCT04916002). In BCC, participants who did not receive hedgehog pathway inhibitors or anti-PD-1/anti-PD-L1 will receive Vidutolimod weekly for 7 doses followed by every 3 weeks for up to 3 years. Cemiplimab is administered every 3 weeks for the same duration.

- Pembrolizumab

New trials have been ongoing for pembrolizumab as monotherapy or in combination with other classes of medications.

In advanced, resectable BCC of the head and neck, pembrolizumab is being evaluated in the neoadjuvant setting in a phase I trial (NCT04323202). Four doses are given prior to surgery, followed by 1 year of adjuvant treatment. A Phase I/II study of pembrolizumab in combination with MDNA11, a long-acting “beta-only” recombinant IL-2, is underway in advanced and metastatic solid tumors, including BCC (NCT05086692). MDNA11 preferentially activates immune effector cells and is also investigated as monotherapy. The safety of a new intra-tumoral medication, MQ710, in combination with pembrolizumab is under clinical investigation for multiple solid tumor histologies including BCC in a phase I trial (NCT05859074). MQ710, a multi-transgene-expressing modified vaccinia virus Ankara-based virotherapy, is initially investigated as monotherapy in the dose escalation phase, then in combination with pembrolizumab in the dose expansion phase.

- Nivolumab

Similar to other checkpoint inhibitors, evaluation of the role of nivolumab in BCC is emerging as a monotherapy or combination. An ongoing phase II trial is investigating the efficacy of nivolumab as monotherapy and in combination with anti-LAG3, relatlimab, or anti-CTLA-4, ipilimumab, in la- or mBCC in the frontline setting or after treatment with hedgehog pathway inhibitor (NCT03521830). The preliminary data presented at the ESMO 2023 annual meeting revealed an objective response rate in 50% among 10 patients with nivolumab monotherapy and 10% among 6 patients who received nivolumab + relatlimab [65]. An ongoing trial of a combination of Intratumoral Talimogene Laherparepvec and nivolumab in refractory cutaneous T-cell lymphoma and other non-melanoma skin cancer is actively recruiting [80]. Talimogene Laherparepvec, a modified herpes virus agent, will first be evaluated as monotherapy. If there is no objective response by week 12, nivolumab will be added [80].

- Vismodegib

Given the frequent dose interruptions with HH inhibitors due to TRAEs, adaptive therapy of vismodegib is an area of active investigation for advanced basal cell carcinoma. NCT05651828 compares four different dosing schedules with treatment tolerance as the endpoint. These dosing schedules include two personalized, intermittent dosing schedules, a fixed intermittent dosing schedule, and a continuous dosing regimen. RADIOSONIC is a phase II study investigating the role of consolidative radiotherapy after obtaining complete response to vismodegib in locally advanced BCC (NCT05561634).

- Novel Intra-Tumoral Therapies

The ARTACUS trial is a phase IB/II study evaluating the efficacy of RP1, an oncolytic virus (genetically modified HSV-1), in solid organ transplant recipients with advanced cutaneous malignancies [76]. The trial is comprised of two parts. In part A, patients will receive 1 × 10^6^ plaque-forming unit (PFU) followed by 1 × 10^7^ PFU. Thereafter, they will continue every two weeks. Part B will enroll lung and heart transplant recipients after determining the safety in the initial part [76]. Another first-in-human study is assessing the safety and tolerability of KB707, a genetically modified HSV-1, in subjects with advanced/refractory solid tumors (NCT05970497).

- Other Novel Therapies

CX-4945, an oral casein kinase 2 (CK2) inhibitor targeting the signaling cascade downstream of Smoothened, was tested in other tumor types and is now investigated in locally advanced and metastatic BCC in a phase I trial. Major eligibility criteria include progression on smoothened inhibitors and receipt of prior radiation [78]. The preliminary analysis showed that 2 of 13 patients had a partial response [77].

START-001 is a phase I/II study to evaluate the efficacy of STAR0602: a selective intravenous T cell receptor (TCR)-targeting, bifunctional antibody-fusion molecule. The cohort includes patients with unresectable, locally advanced, or metastatic solid tumors that are antigen-rich (NCT05592626). Oral PD-L1 inhibitors are newly investigated in this landscape. INCB099318 is currently in a phase I multicenter study in patients who had disease progression after standard therapies (NCT04272034). No dose-limiting toxicities occurred; among the enrolled 32 patients, one had grade 3 treatment-emerged adverse event, and 10 patients discontinued therapy due to disease progression [79].

## 7. Conclusions

The development of systemic treatments for advanced BCC is an emerging and evolving field. The current standard of care for locally advanced/metastatic disease includes HH inhibitors, vismodegib or sonidegib, which have a toxicity profile that could be severe. In addition, for patients whose disease has become resistance to this class of medication, or for those ineligible or intolerant of HH inhibitors, cemiplimab immunotherapy is recommended as second-line standard treatment. Novel therapies to address treatment resistance are under investigation. In cases of HH inhibitor resistance, targeting the downstream molecule, CK2, is promising per preliminary results, but more data are yet to be evaluated [77]. In addition, the combination of HH inhibitor and immunotherapy might also be a solution to overcome this challenge or primary or secondary resistance.

The ongoing clinical trials evaluating immunotherapy in advanced BCC highlight the growing need for and interest in this class of therapy. However, it is critical to further characterize the mechanism of resistance to immune checkpoint inhibitors and to identify biomarkers that inform prognosis and that are predictive of response. Prior research did not reveal the significance of high TMB threshold or PD-L1 expression in this context. However, the downregulation of MHC-I expression is an attractive biomarker in this context for which more studies are needed to prove this hypothesis. Intra-tumoral injections, in conjunction with immunotherapy, is an interesting aspect to be further explored. Trials are still in early phases, and more data are needed for safety and efficacy. Finally, evaluating the role of immune checkpoint and hedgehog inhibitors in the neoadjuvant and adjuvant settings is essential, and trials are ongoing to evaluate the potential benefit of combination therapy for BCC.

## Figures and Tables

**Table 2:** Ongoing Clinical Trials in Advanced BCC.

NCT	Study Title	Phase	Intervention	Status
04679480	A Prospective, Open, Single-arm, Single Center, Phase II Trial to Assess the Efficacy of Anti-PD1 Antibody in Combination with Pulsed Hedgehog Inhibitor in Advanced Basal Cell Carcinoma	II	Cemiplimab Sonidegib	Recruiting
03521830	Nivolumab Alone or Plus Relatlimab or Ipilimumab for Patients with Locally Advanced Unresectable or Metastatic Basal Cell Carcinoma	II	Nivolumab Ipilimumab Relatlimab	Recruiting [65]
05651828	Adaptive Therapy of Vismodegib in Advanced Basal Cell Carcinoma	I	Vismodegib	Recruiting
05929664	Neoadjuvant REGN2810 (Cemiplimab) in Cutaneous Basal Cell Carcinoma of the Head and Neck	II	Cemiplimab	Recruiting
04806646	A Phase II, Open-label Study Improving Compliance and Time of Treatment After Obtaining Complete Response Through a Tailored Schedule of Sonidegib in Locally Advanced Basal Cell Carcinomas (BCC)-the SONIBEC Trial	II	Sonidegib	Recruiting
05561634	Evaluation of Radiotherapy After Complete Response to Sonic Hedgehog Pathway Inhibitors in Patients with Locally Advanced Basal Cell Carcinoma: A Prospective Multicenter Study	II	Vismodegib Radiotherapy	Not yet recruiting
04349436	An Open-Label, Multicenter, Phase 1B/2 Study of RP1 in Solid Organ and Hematopoietic Cell Transplant Recipients with Advanced Cutaneous Malignancies	IB/II	RP1 (Intra-tumoral injection)	Recruiting [76]
04916002	A Multicenter, Open-label, Phase 2 Study of Intratumoral Vidutolimod (CMP-001) in Combination with Intravenous Cemiplimab in Subjects with Selected Types of Advanced or Metastatic Cancer	II	Vidutolimod (CMP-001, intra-tumoral) Cemiplimab	Recruiting
05970497	A Phase 1, Open-Label, Multi-Center, Dose Escalation and Expansion Study of KB707 in Subjects with Locally Advanced or Metastatic Solid Tumor Malignancies	I	KB707 (HSV1, Intra-tumoral)	Recruiting
05859074	A First-In-Human Phase I, Open Label, Safety and Tolerability Study of Escalating Multiple Doses of Intratumoral MQ710, a Multi-Transgene Expressing Modified Vaccinia Virus Ankara-Based Virotherapy, Alone and in Combination with the Systemic Checkpoint Inhibitor Pembrolizumab in Solid Tumors	I	MQ710 Pembrolizumab	Recruiting
05086692	A Phase 1/2 Open Label, Dose Escalation and Expansion Study of MDNA11, IL-2 Superkine, Administered Alone or in Combination with Immune Checkpoint Inhibitor in Patients with Advanced Solid Tumors	I/II	MDNA11 Pembrolizumab	Recruiting
05592626	A Phase 1/2, First-in-Human, Open-Label, Dose Escalation and Expansion Study of STAR0602, a Selective T Cell Receptor (TCR) Targeting, Bifunctional Antibody-fusion Molecule, in Subjects with Unresectable, Locally Advanced, or Metastatic Solid Tumors That Are Antigen-rich (START-001)	I/II	STAR0602	Recruiting
03897036	A Phase I, Multi-Center, Open-Label, Treatment Duration Increment, Expansion, Safety, and Pharmacodynamic Study of CX-4945 Administered Orally Twice Daily to Patients with Advanced Basal Cell Carcinoma	I	CX-4945	Not recruiting [77,78]
04323202	A Phase 1B, Single Arm Study of Neoadjuvant-Adjuvant Pembrolizumab in Resectable Advanced Basal Cell Carcinoma of the Head and Neck to Assess for Pathologic Responses in the Tumor Microenvironment	I	Pembrolizumab	Not recruiting
04272034	A Phase 1 Study Exploring the Safety, Tolerability, Pharmacokinetics, and Pharmacodynamics of INCB099318 in Participants with Select Advanced Solid Tumors	I	INCB099318	Not recruiting [79]
02978625	A Phase II Study of Talimogene Laherparepvec Followed by Talimogene Laherparepvec + Nivolumab in Refractory T Cell and NK Cell Lymphomas, Cutaneous Squamous Cell Carcinoma, Merkel Cell Carcinoma, and Other Rare Skin Tumors	II	Talimogene Laherparepvec Nivolumab	Not recruiting [80]
03775525	A Phase I/Ib, Multicenter, Open-label, Dose-Escalation, Safety, Pharmacodynamic and Pharmacokinetic Study of GZ17-6.02 Monotherapy and in Combination with Capecitabine, Given Orally on a Daily Schedule in Patients with Advanced Solid Tumors or Lymphoma	I	GZ17-6.02 Capecitabine	Not recruiting
